# Circulating and tumor-infiltrating arginase 1-expressing cells in gastric adenocarcinoma patients were mainly immature and monocytic Myeloid-derived suppressor cells

**DOI:** 10.1038/s41598-020-64841-4

**Published:** 2020-05-15

**Authors:** WeiHong Ren, XuRan Zhang, WenBo Li, Qian Feng, HuiJie Feng, Yan Tong, Hao Rong, Wei Wang, Dai Zhang, ZhenQiang Zhang, ShiChun Tu

**Affiliations:** 1 Department of Laboratory Medicine, The First Affiliated Hospital of Henan University of Chinese Medicine, No.19 Renmin Road, Zhengzhou, Henan Province China; 2 Immunology Laboratory of Chinese Medicine, Henan University of Chinese Medicine, No.156 Jinshui East Road, Zhengzhou, Henan Province China; 3 Neurodegenerative Disease Center, Scintillon Institute, San Diego, CA 92121 USA

**Keywords:** Immunoediting, Gastric cancer, Tumour immunology

## Abstract

Myeloid-derived suppressor cells (MDSCs) are a group of heterogeneous cells derived from immature myeloid cells (IMCs). MDSCs are known to play important roles in tumor immune evasion. While we know that there are a large number of circulating and tumor-infiltrating MDSCs existing in gastric cancer (GC) patients, the phenotypic characteristics and arginase 1 (ARG1) expression levels of these MDSCs remain very unclear. In our study, flow cytometric analysis of circulating MDSCs from 20 gastric adenocarcinoma (GAC) patients found that ≥80% ARG1-expressing MDSCs were mainly early-stage MDSCs (HLA-DR^−^CD33^+^CD14^−^CD15^−^MDSCs). In addition, our investigation showed that tumor-infiltrating MDSCs from 6 GAC patients consisted of >35% ARG1-expressing naïve MDSCs (HLA-DR^−^CD33^−^CD11b^−^CD14^−^CD15^−^MDSCs), >15% early-stage MDSCs and >40% monocytic MDSCs (HLA-DR^−^CD14^+^MDSCs). This preliminary study describes the phenotypic characteristics and ARG1 expression levels of MDSCs from GAC patients and shows that circulating and tumor-infiltrating ARG1-expressing cells were mainly immature and monocytic MDSCs, which provides information to better understand the mechanisms that allow gastric cancer cells to evade the immune system.

## Introduction

Myeloid-derived suppressor cells (MDSCs) are myeloid-derived heterogeneous cell populations composed mainly of immature dendritic cells, macrophages and granulocytes. An important characteristic of these cells is the production of suppressive factors such as arginase 1 (ARG1), inducible nitric oxide and (iNOS; also known as NOS2), reactive oxygen and reactive nitrogen species. MDSCs also modulate the production of various cytokines, which are potent suppressors of various T-cell functions^1–4^. MDSCs are known to play important roles in human disease^5–7^, and thus have become an important therapeutic target^8,9^.

In mice, MDSCs are characterized by the co-expression of the myeloid lineage differentiation antigens, CD11b and Gr1^10,11^. In humans, the specific identification of MDSCs is a complex and challenging task^12^. In human peripheral blood mononuclear cells (PBMCs), MDSCs are most commonly defined as CD11b^+^, CD33^+^ and HLA-DR^−/low^ cells^2,13^. CD11b^+^CD14^−^CD15^+^ and CD11b^+^CD14^−^CD66b^+^MDSC are defined as polymorphonuclear MDSCs (PMN-MDSCs)^14,15^, while HLA-DR^−/low^CD11b^+^CD14^+^CD15^-^MDSCs are defined as monocytic MDSCs (M-MDSCs). Lin^-^(including CD3, CD14, CD15, CD19, CD56) HLA-DR^-^CD33^+^MDSCs are defined as early-stage MDSCs (eMDSCs)^15,16^. The immunophenotypes of MDSCs induced by different types of tumors show variability. Many such phenotypes have been reported, including HLA-DR^-^CD33^+^MDSC^17–19^, HLA-DR^-/low^CD14^+^MDSC^20–22^, Lin^-^HLA-DR^-^CD33^+^MDSC^23–25^.

A number of studies relating to gastric cancer (GC) have reported MDSCs phenotype markers, including CD11b^+^, CD33^+^, CD14^+^, CD15^+^and HLA-DR^-^^26,27^. However, there is limited research data on this point at present and there has been no clear explanation of the phenotypic features of MDSCs or the expression level of ARG1 in MDSCs.

In this study, we first analyzed by flow cytometry the phenotype and the ARG1 expression level in MDSCs from peripheral blood and cancerous tissue of gastric adenocarcinoma (GAC) patients. Next, we determined the relative expression levels of ARG1/NOS2 in those circulating and tumor-infiltrating MDSCs by qRT-PCR and Western blotting. These findings provide clues for the further elucidation of the exact mechanism by which gastric cancer cells induce the generation of MDSCs, and how the immune function of these key cells is activated.

## Materials and methods

### Clinical sample collection

The study was conducted in accordance with the 1964 Declaration of Helsinki. The project was approved by the Medical Ethics Committee of the First Affiliated Hospital of Henan University of Chinese Medicine (Zhengzhou, China) (protocol number 2016HL-085), and all individual participants included in the study gave written informed consent. Backgrounds of gastric cancer patients and volunteers are described in Supplementary Table 1. All patients with GAC were first diagnosed and enrolled without radiotherapy or chemotherapy. From November 2016 to March 2017, 6 GAC tissue specimens were collected at the general surgery department of the First Affiliated Hospital of Henan University of Chinese Medicine. In each case, specimens of cancerous tissues and paracancerous tissues (5 cm from the edge of the cancer) were taken. Tissue specimens were collected in sterile test tubes containing RPMI1640 culture medium. Simultaneously, peripheral blood samples were collected from 20 GAC patients prior to surgery. 20 ml blood sample were placed into EDTA.K_2_ anticoagulant test tubes and immediately sent to the laboratory for analysis.

### Preparation of mononuclear cells suspension from cancerous or paracancerous tissue

The cancerous and paracancerous tissue masses were cut into 2–4 mm^3^ pieces. Fat, fibrous tissue and necrotic tissue was removed. Tissue masses were then transferred into a GentleMACS Dissociator. (Miltenyi Biotec, Bergisch-Gladbach, Germany). These techniques were carried out in accordance with the manufacturer’s instructions. Following homogenization, the sample was centrifuged at 300 g for 7 min and the pellet was collected. The pellet was resuspended in 20 ml of RPMI1640 and any larger tissue masses were picked out using a sterile pipette tip. This suspension was then placed into a 70μm MACS SmartStrainer, and the column rinsed with 20 ml of RPMI1640 to gather the suspension on the filter. The column was then centrifuged at 300 g for 7 min, the supernatant discarded, and the cells were resuspended in preparation for subsequent experiments.

The prepared single tumor cell suspension was carefully added to 2 ml of human organ tissue mononuclear cells separation solution (HaoYang Biotechnology company, TianJin, China) and centrifuged at 300–500 g for 15 minutes. Centrifugation separated the preparation into four layers. The top layer was fetal bovine serum, the second layer consisted of loop ivory mononuclear cells, the third layer was transparent separation medium and the fourth layer consisted of histocytes. Cells in the second layer were collected into a test tube and added to 4–5 ml of phosphate buffered saline (PBS). This mixture was then blended thoroughly and centrifuged at 300–500 g for 30 minutes. Cells were washed twice with PBS and then collected.

### Preparation of PBMCs

10 ml of fresh peripheral blood, which had been anticoagulated with EDTA.K_2_, was mixed with RPMI1640 culture medium at a ratio of 1:1. Next, 4 ml of human peripheral blood lymphocyte separation solution was added to a 15 ml centrifuge tube, the diluted peripheral blood mixture was carefully layered onto the cell separation solution (at a ratio of 2:1) and the tube centrifuged at 1000 g for 15 minutes at 18 °C. Following centrifugation, the mixture had separated into the following four layers: the first layer (at the top) consisted of plasma, the second consisted of loop ivory lymphocytes, the third was transparent separation medium and the fourth consisted of erythrocytes. The cells in the second layer were collected and placed into a 15 ml tube. Cells were washed in AIM-V (Grand Island Biological Company, New York, NY, USA) culture medium and centrifuged at 600 g for 7 minutes. The supernatant was then discarded and the pelleted cells resuspended in AIM-V. The washing step was repeated two further times and resulted in the isolation of mononuclear cells.

### Sorting HLA-DR^-/low^ mononuclear cells by means of magnetic beads

To obtain HLA-DR^-/low^ mononuclear cells from tumor tissue and peripheral blood, we took an appropriate amount of mononuclear cell suspension from cancerous or paracancerous tissue or peripheral blood mononuclear cells and washed in pre-cooled autoMACS® Running Buffer (Miltenyi Biotec, Bergisch-Gladbach, Germany). Following resuspension, HLA-DR^-/low^ mononuclear cells were isolated by negative sorting (anti-HLA-DR magnetic bead, LS column and a MACS Magnetic shelf) (Miltenyi Biotec). After sorting, the number of living cells was calculated by trypan blue staining. HLA-DR^-/low^ mononuclear cells were then fluorescently labelled with anti-HLA-DR-PE(20 μl/10^7^cells) (BD Biosciences, Franklin Lakes, NJ, USA) and a CantoII flow cytometer(BD Biosciences) was used to analyze the purity of the HLA-DR^-/low^ cells.

### Sorting HLA-DR^-^CD33^+^MDSCs by means of magnetic beads

To separate the CD33^+^ population, HLA-DR^-^ cells were co-incubated with CD33 microbeads, and HLA-DR^-^CD33^+^MDSCs were collected using a Positive Selection program. Cell viability of sorted HLA-DR^-^CD33^+^MDSCs was analyzed using trypan blue staining; the purity was then validated on a FACS Canto II flow cytometer using an APC-conjugated anti-CD33 antibody (BD Biosciences, USA).

### Sorting CD8^+^T cells by means of magnetic beads

To obtain CD8^+^T cells, PBMCs from healthy donors were washed with precooled autoMACS® Running Buffer (Miltenyi Biotec, Germany), incubated with anti-CD8 microbeads, and CD8^+^T cells were separated on a autoMACS® separator using the Depletion program following manufacturer’s instructions. Sorted CD8^+^T cells were labeled with CFSE (3 μM, Sigma)

### Analysis of myeloid cells by means of flow cytometry

In order to label peripheral blood MDSCs with a fluorescent antibody (BD Biosciences), 100 μl of peripheral blood was added to a test tube containing fluorescent labeled antibody or isotype control, mixed gently, and incubated for 20 minutes without light at 4 °C. Next, 400 μl of erythrocyte lysis buffer was added and mixed gently prior to incubation for 15 minutes without light at 25 °C. In order to label tumor-infiltrated MDSCs with a fluorescent antibody, 100 μl of prepared cancerous or paracancerous tissue mononuclear cell suspension (1 × 10^6^/ml) was added to a test tube containing fluorescent labeled antibody or isotype control, mixed gently, and incubated for 20 minutes without light at 4 °C.

In order to remove uncombined fluorescent antibodies, samples were centrifuged at 1000 g or 5 minutes and the supernatant was discarded. The pellet was then washed twice with PBS. The labeled cells were resuspended with 300 μl of flow cytometry (FCM) buffer solution prior to detection. Flow cytometry (Canto II type, BD FACSDiva^TM^ analysis software) was then used to analyze the immunophenotype of circulating and tumor-infiltrating MDSCs that had been labeled with fluorescent antibody.

### Detecting intracellular ARG1 in MDSCs subgroups by means of flow cytometry

Immune cells in peripheral blood or tumor tissue were labeled with anti-HLA-DR-PE/anti-CD33-APC/anti-CD11b-APC-Cy7/anti-CD14-PerCP-Cy5.5/anti-CD15-PE-Cy7 fluorescent antibodies. At the same time an isotype control (mouse IgG2b-PE/mouse IgG1-APC/rat IgG2b APC-CY7/mouse IgG2a-PerCP-Cy5.5/mouse IgM-PE-CY7) (BD Biosciences) was prepared. Labeled cells were centrifuged at 300 g for 15 minutes at 18 °C and the supernatant was discarded. The pellet was then gently mixed with 100 μl of cytomembrane punching reagent 1 (Beckman Coulter Inc., Brea, CA, USA), which was mixed gently, and incubated for 15 minutes without light at 25 °C. Cells were then washed with normal saline (NS) and centrifuged at 300 g for 5 minutes. The supernatant was then discarded and the pellet mixed with 100 μl of cytomembrane punching reagent 2, and incubated for 5 minutes without light at 25 °C. Then, 5 μl of ARG1-FITC was added and incubated for 15 minutes without light at 25 °C, simultaneous with an isotype control (mouse IgG1 FITC). Next, 1 ml of NS was added and the mixture centrifuged at 300 g for 5 minutes. The supernatant was then discarded and the labelled cells resuspended in 500 μl of FCM buffer solution for detection.

### Reverse-transcription polymerase chain reaction (RT-PCR)

An RNA Extraction Kit (Thermo Fisher Scientific, Inc., Waltham, MA, USA) was used to extract RNA from Lin^-^HLA-DR^-^cells derived from tumor tissue or peripheral blood. A reverse-transcription was then performed to reverse transcribe mRNA into cDNA. Primers were synthesized by Shanghai Biological Engineering Co. LTD. Primer sequences were determined using NCBI BLAST software (please refer to Table 1).

**Table 1:** ARG1, NOS2 and GAPDH primer sequences.

Target gene	Primer sequence
ARG1	Forward primer 5′ACTTAAAGAACAAGAGTGTGATGTG3′
	Reverse primer 5′GTCCACGTCTCTCAAGCCAA3′
NOS2	Forward primer 5′TCCCGAAGTTCTCAAGGCAC3′
	Reverse primer 5′CATAGCGGATGAGCTGAGCA3′
GAPDH	Forward primer 5′CGAGATCCCTCCAAAATCAA3′
	Reverse primer 5′TTCACACCCATGACGAACAT3′

Next, we used a real-time fluorescence quantification kit to amplify cDNA on an ABI 7500 system (Applied Biosystems, Carlsbad, CA, USA) to calculate the expression level of each target gene relative to an internal reference gene using the 2^−ΔΔCT^ method.

### Western blot analysis

HLA-DR^-/low^ mononuclear cells isolated from peripheral blood or tumor tissue were lyzed using RIPA lysate buffer (50 mM Tris-HCl (pH 7.4), 1% (v/v) Triton X-100, 1 mM EDTA, 1 mM leupeptin, 1 mM phenylmethylsulfonyl fluoride, 10 mM NaF, 1 mM Na_3_VO_4_). Lyzed cells were then centrifuged at 13,000 g for 15 minutes. Concentrations of extracted protein were then determined using a Bicinchoninic Acid (BCA) kit (Sigma-Aldrich Co., St Louis, MO, USA). For each sample, 60 μg of protein was then separated by SDS-polyacrylamide gel (SDS-PAGE) electrophoresis, and then transferred to PVDF membranes. Membranes were then incubated overnight at 4 °C with anti-ARG1-Ab, anti-NOS2-Ab and anti-β-actin (1:5000) antibodies. The PVDF membrane was then washed 4 times with 0.01 M PBS (5 min per wash) and then incubated for 2 hours at 25 °C with goat anti-mouse IgG-HRP (Santa Cruz Biotechnology, Santa Cruz, CA, USA). After color rendering by o-Phenylenediamine (OPD) and 3,3’,5,5’-Tetramethylbenzidine (TMB), the PVDF membrane was scanned by a SYSTEM GelDoc XR + scanner (Bio-Rad Laboratories, Inc., Hercules, CA, USA). The net density and transparent ratio of each band was then analyzed by Image J software.

### Functional analysis of MDSCs

Sorted HLA-DR^-^CD33^+^MDSCs were co-cultured with CD8^+^T cells (MDSCs:T cells = 1:4) *in vitro* in RPMI-1640 complete medium supplemented with 2 mM L-glutamine, 10 mM HEPES, 20 mM 2-ME, 10% fetal bovine serum, penicillin/streptomycin, and 40 ng/ml hGM-CSF, at 37 °C in a humidified 5% CO_2_ incubator. T cell proliferation was induce by anti-CD3/CD28 stimulation beads (Invitrogen, Carlsbad, CA). After 24 hour of co-culture, T cell proliferation was analyzed on a CantoII flow cytometer(BD Biosciences), after 72 hour of co-culture supernatants were analyzed for IFNγ levels on Luminex^®^200TM (luminex company, Austin, Texas, USA).

### Statistical analysis

Flow cytometry and real-time PCR were performed in triplicate, and each experiment was repeated at least three times. All western blot images and semi-quantitative qRT-PCR results are representative of at least three independent experiments. Data are presented as mean ± standard deviation (SD) of at least three independent experiments. *T*-tests were used to compare the differences between two groups, Paired/Wilcoxon matched-pairs signed rank test or unpaired/Mann–Whitney tests were used to examine the differences within groups or between groups, respectively. All P values were 2 sided and considered to be statistically significant at *p* < 0.05 (*p < 0.05, **p < 0.01, as indicated in the figures and legends).

### Ethics approval and consent to participate

The study was conducted in accordance with the 1964 Helsinki Declaration. All procedures performed and all clinical data obtained in the present study involving human participants were reviewed and approved by the Medical Ethics Committee of the First Affiliated Hospital of Henan University of Chinese Medicine (protocol number 2016HL-085). All individual participants included in the study gave written informed consent.

### Open Access

This article is distributed under the terms of the Creative Commons Attribution License which permits any use, distribution, and reproduction in any medium, provided the original author(s) and the source are credited.

## Results

### The purity of HLA-DR^-/low^ mononuclear cells

**Figure 1:**
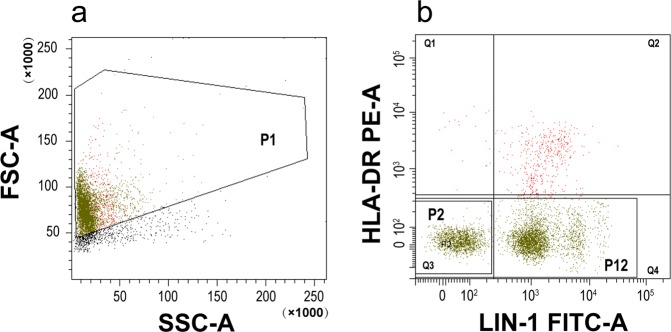
Purity of HLA-DR^-/low^ monocnuclear cells. HLA-DR^-/low^ monocnuclear cells were sorted by magnetic beads. The purity of these cells was 97.6%, as determined by flow cytometry (Q3 + Q4 = 97.6%). *Abbreviations: P1, population1 (HLA-DR*^*-/low*^ *cells were sorted by magnetic beads); P2, population2 (HLA-DR*^*-/low*^*lin*^*-/low*^ *cells); P12, population12 (HLA-DR*^*-/low*^ *cells)*.

We found the purity of HLA-DR^-/low^ mononuclear cells sorted by magnetic beads to be more than 95%, as determined by flow cytometry; The proportion of Lin^-^HLA-DR^-/low^ cells was more than 60% (Fig. 1).Trypan blue staining showed the total number of living cells to be more than 90% and was sufficient for all subsequent experiments.

### ARG1-expressing cells in peripheral blood of GAC patients were mainly early-stage MDSCs

**Figure 2:**
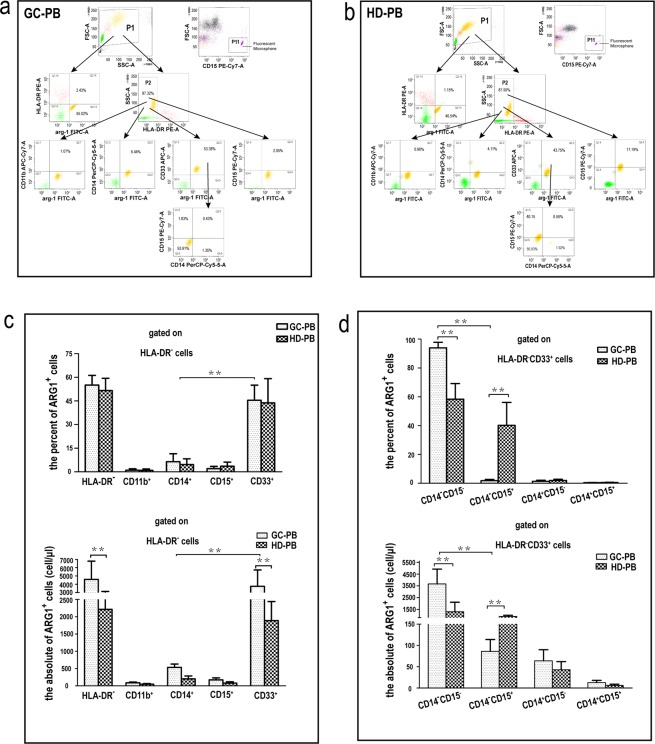
Percentage and count of ARG1-expressing cells in different immunophenotypes of circulating-MDSCs. (**a**) Flow cytometry chart of ARG1-expressing MDSCs in the peripheral blood of gastric cancer patients (GC-PB). Cells were first gated based on the expression of HLA-DR. Subsequently, the HLADR ^-/low^ population were gated based on the co-expression of ARG1 and CD11b, ARG1 and CD14, ARG1 and CD15, ARG1 and CD33; the CD33^+^ARG1^+^ cells were gated based on the expression of CD14 and CD15. An isotype control was also run (Supplementary Fig. 1). (**b**) Flow cytometry chat of ARG1-expressing cells in the peripheral blood of healthy donors (HD-PB). Flow cytometry showing different MDSC phenotypes in GC-PB or HD-PB. An isotype control was also run (Supplementary Fig. 1). (**c**) Gated on HLA-DR^-/low^ cells, the percentage and count of ARG1-expressing cells in CD33^+^ or CD14^+^ or CD15^+^ or CD11b^+^ MDSC subsets of peripheral blood. (**d**) Gated on HLA-DR^-^CD33^+^MDSCs, the percentage and count of ARG1-expressing cells in CD14^+^CD15^-^ or CD14^+^CD15^+^ or CD14^-^CD15^+^ or CD14^-^CD15^-^ MDSC subsets of peripheral blood. Values were mean ± SEM (n = 20), **p < 0.01. *Paired /Wilcoxon matched-pairs signed rank test or unpaired/Mann–Whitney tests*. *Abbreviations: P11, population11 (CaliBRITE fluorescent microsphere)*.

To study the phenotype and the ARG1 expression level of circulating myeloid cells from GAC patients, we collected peripheral blood samples from 20 GAC patients and 20 age-matched healthy volunteers. We used flow cytometry to analyze the phenotype and number of MDSCs and then qRT-PCR and western blotting to determine the expression level of ARG1 mRNA and protein, respectively, in different phenotypes of MDSCs. We first gated PBMCs based on the expression of HLA-DR and ARG1 (Fig. 2a,b). In the peripheral blood of healthy volunteers, HLA-DR^-/low^ cells were the predominant cell type, and ARG1 expression levels were high (Fig. 2b). Compared to healthy volunteers, the number of HLA-DR^-/low^ cells was significantly higher (7315 ± 2791/μl *vs* 4287 ± 1318/μl) (*p* < 0.01), and the proportion of ARG1-expressing cells was not significantly higher (55.02 ± 7.53% *vs* 43.75 ± 11.15%) (*p* > 0.05) in the peripheral blood of GAC patients (Fig. 2c).

To further elucidate the cellular phenotypic characteristics of ARG1-expressing cells, we determined the immunophenotype of HLA-DR^-/low^ cells in the peripheral blood of GAC patients after labeling them with CD33/CD11b/CD14/CD15 antibodies. Results indicated that the ARG1-expressing MDSCs were mainly HLA-DR^-^CD33^+^MDSCs (53.38 ± 10.40% vs 43.75 ± 8.31%) (Fig. 2c). In addition, a small number of HLA-DR^-^CD14^+^MDSCs (6.44 ± 1.56% vs 4.11 ± 1.32%), and HLA-DR^-^CD15^+^MDSCs (2.05 ± 0.52% vs 17.19 ± 3.25%) could also express ARG1 (*p* < 0.01) (Fig. 2c). Further immunophenotyping of HLA-DR^-^CD33^+^MDSCs indicated that ARG1-expressing MDSCs were mainly CD14^-^CD15^-^MDSCs (early-stage MDSCs) (93.91 ± 3.42% vs 56.93 ± 3.25%) (*p* < 0.01), other subtypes expressed only trace levels of ARG1 (*p* < 0.01) (Fig. 2d).

### ARG1-expressing cells in tumor tissue were mainly immature and monocytic MDSCs

**Figure 3:**
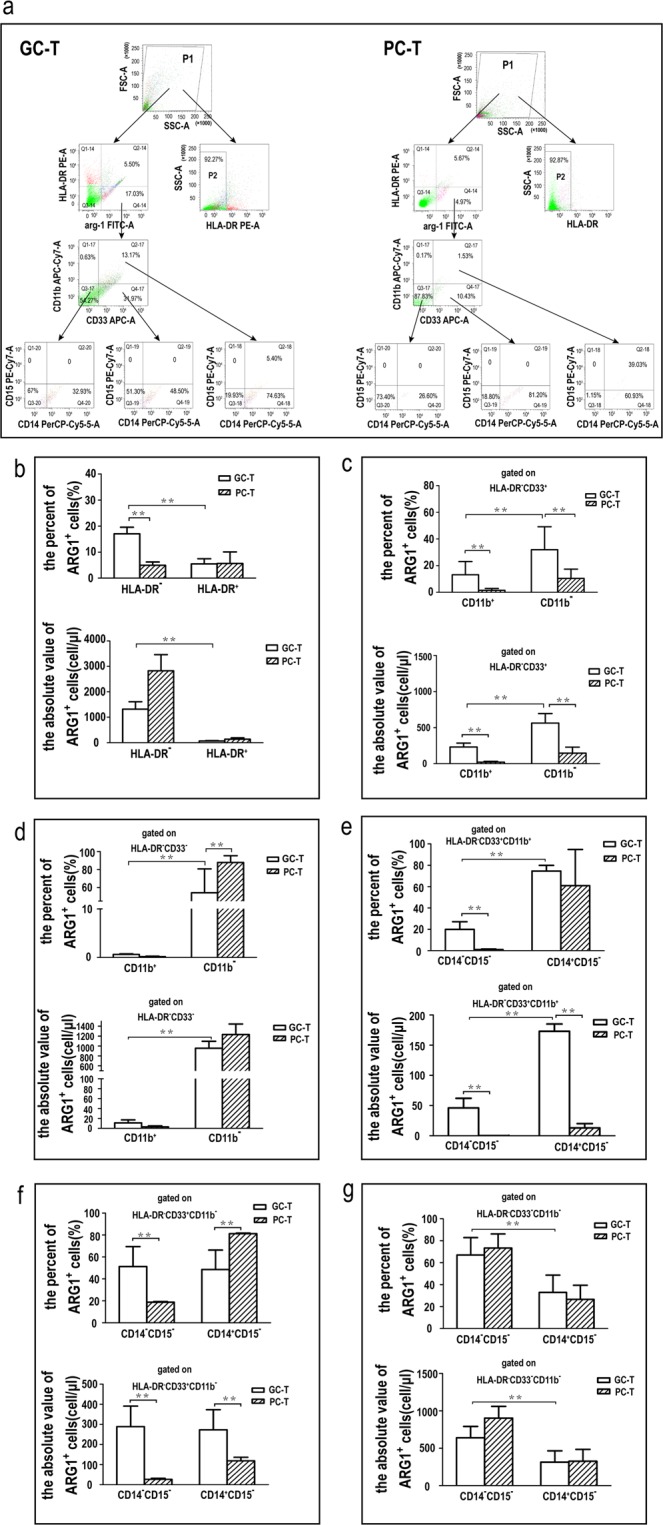
Percentage and count of ARG1-expressing cells in different immunophenotype tumor-infiltrating MDSCs. (**a**) Flow cytometry chart of ARG1-expressing MDSCs in gastric cancer tissue (GC-T) and paracancerous tissues (PC-T). Cells were first gated based on the expression of HLA-DR and ARG1. Subsequently, the HLA-DR ^-/low^ ARG1^+^ population were gated based on the expression of CD11b and CD33. The HLA-DR^-^CD33^+^CD11b^-^, HLA-DR^-^CD33^+^CD11b^+^ and HLA-DR^-^CD33^-^CD11b^-^ population were gated based on the expression of CD14 and CD15. An isotype control was also run (Supplementary Fig. 2). (**b**) The percentage and count of ARG1-expressing HLA-DR^-^ mononuclear cells in GC-T or PC-T. (**c**) Gated on HLA-DR^-^CD33^+^, the percentage and count of ARG1-expressing CD11b^+^ or CD11b^-^ MDSC subsets in GC-T or PC-T. (**d**) Gated on HLA-DR^-^CD33^-^, the percentage and count of ARG1-expressing CD11b^+^ or CD11b^-^ MDSC subsets in GC-T or PC-T. (**e**) Gated on HLA-DR^-^CD33^+^CD11b^+^, the percentage and count of ARG1-expressing CD14^+^CD15^-^ or CD14^-^CD15^-^MDSC subsets in GC-T or PC-T. (**f**) Gated on HLA-DR^-^CD33^+^CD11b^-^, the percentage and count of ARG1-expressing CD14^+^CD15^-^ or CD14^-^CD15^-^MDSC subsets in GC-T or PC-T. (**g**) Gated on HLA-DR^-^CD33^-^CD11b^-^, The percentage and count of ARG1-expressing CD14^+^CD15^-^ or CD14^-^CD15^-^MDSC subsets in GC-T or PC-T. Values were mean ± SEM (n = 6), **p < 0.01. *Paired /Wilcoxon matched-pairs signed rank test or unpaired/Mann–Whitney tests*.

In order to observe the phenotypic characteristics and the ARG1 expression level of tumor-infiltrating MDSCs from GAC patients, we collected cancerous and paracancerous tissues from 6 GAC patients to prepare mononuclear cell suspensions. We then determined their immunophenotype by using BD FCSCantoTM-II flow cytometry. Data indicated that most of the mononuclear cells in cancerous and paracancerous tissues were HLA-DR^-/low^ cells (92.27 ± 4.72% *vs* 92.87 ± 4.06%) (*p* > 0.05) (Fig. 3a). However, HLA-DR^-/low^ cells with high expression levels of ARG1 were significantly more common in cancerous tissues than in the paracancerous tissue (17.03 ± 2.50% *vs* 4.97 ± 1.40%) (*p* < 0.01) (Fig. 3a,b).

Compared to paracancerous tissue, further immunophenotyping of HLA-DR^-/low^ cells indicated that ARG1-expressing cells in cancerous tissues were CD33^+^CD11b^-^MDSCs (31.97 ± 10.90% *vs* 10.40 ± 4.78%) (*p* < 0.01) (Fig. 3a,c), CD33^+^CD11b^+^MDSCs (13.17 ± 7.04% *vs* 1.53 ± 0.88%) (*p* < 0.01) (Fig. 3a,c) and CD33^-^CD11b^-^MDSCs (54.27 ± 26.50% *vs* 87.83 ± 7.51%) (*p* < 0.01) (Fig. 3a,c). However, only a few CD33^-^CD11b^+^MDSCs in tumor tissues expressed ARG1 (0.63 ± 0.29% *vs* 0.17 ± 0.12%) (*p* > 0.05) (Fig. 3a,d).

We could also subdivide CD33^+^CD11b^+^MDSCs into CD14^+^CD15^-^ monocytic MDSCs (74.63 ± 5.78% *vs* 60.93 ± 15.85%) (*p* > 0.05) and CD14^-^CD15^-^early-stage MDSCs (19.93 ± 5.60% vs 1.15 ± 0.58%) (*p* < 0.01) (Fig. 3a,e). We further subdivided CD33^+^CD11b^-^MDSCs into CD14^+^CD15^-^monocytic MDSCs (48.50 ± 12.43% *vs* 81.20 ± 4.20%) (*p* < 0.01) and CD14^-^CD15^-^early-stage MDSCs (51.30 ± 12.60% vs 18.80 ± 4.20%) (*p* < 0.01) (Fig. 3a,f). CD33^-^CD11b^-^MDSCs can also be sub-divided into CD14^+^CD15^-^monocytic MDSCs (32.93 ± 4.56% *vs* 26.60 ± 4.23%) (*p* > 0.05) and CD14^-^CD15^-^naïve MDSCs (67.00 ± 8.60% vs 73.40 ± 9.35%) (*p* > 0.05), but the percent and the absolute value of naïve MDSCs were significantly more than those of monocytic MDSCs (*p* < 0.01) (Fig. 3a,g).

### ARG1 and NOS2 were highly expressed in circulating and tumor-infiltrating HLA-DR^-/low^ mononuclear cells from GAC patients

**Figure 4:**
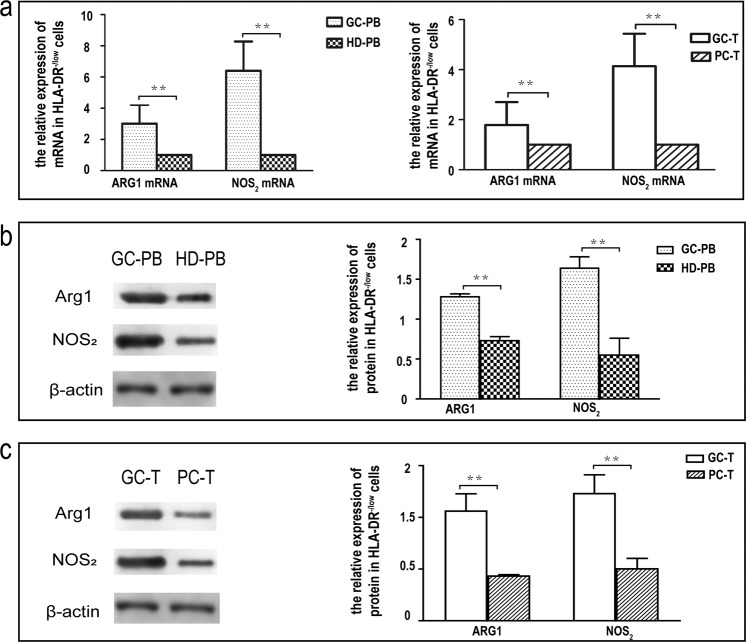
ARG1 and NOS2 expression levels in circulating and tumor-infiltrating HLA-DR-/low mononuclear cells. (**a**) Analysis of ARG1 and NOS2 expression levels in circulating and tumor-infiltrating HLA-DR^-/low^ mononuclear cells by means of flow cytometry. Figure 4a (left): The relative expression levels of ARG1 and NOS2 mRNA in the circulating HLA-DR^-/low^ mononuclear cells (n = 20). Figure 4a (right): The relative gene-expression levels of ARG1 and NOS2 mRNA in the tumor-infiltrating HLA-DR^-/low^ mononuclear cells (n = 6). (**b**,**c**) Analysis of ARG1 and NOS2 expression level in circulating and tumor-infiltrating HLA-DR^-/low^ mononuclear cells by means of western blot. Normalized relative expression of western blot bands was calculated by setting one band as 1. Data are representative of three biological replicates. (**b**) The relative expression levels of ARG1 and NOS2 protein in the circulating HLA-DR^-/low^ mononuclear cells (n = 20). (**c**) The relative expression levels of ARG1 and NOS2 protein in the tumor-infiltrating HLA-DR^-/low^ mononuclear cells (n = 6). Values were mean ± SEM, **p < 0.01 determined by Student’s *t-test*. *Full length blots of b and c are provided in* Supplementary Fig. 2*. Abbreviations: GC-PB, gastric cancer-peripheral blood; HD-PB, healthy donor-peripheral blood. GC-T, gastric cancer-tissue; PC-T, paracancerous tissues*.

To further verify the results derived from flow analysis, we investigated ARG1 and NOS2 expression in HLA-DR^-/low^ mononuclear cells in peripheral blood and cancerous tissues from GAC patients. First, HLA-DR^-/low^ mononuclear cells were sorted from peripheral blood and a mononuclear cell suspension from tumor tissue. Then, the relative expression levels of ARG1 mRNA and NOS2 mRNA were detected by qRT-PCR, and the relative expression levels of ARG1 and NOS2 protein were detected by western blotting. The relative expression levels of ARG1 mRNA and NOS2 mRNAs in HLA-DR^-/low^ mononuclear cells from the GAC patients’ peripheral blood was 3.01 times and 6.4 times higher respectively, than those of healthy volunteers (*p* < 0.01) (Fig. 4a). The relative expression levels of ARG1 and NOS2 protein were increased by factors of 3.89 and 3.49, respectively, (*p* < 0.01) (Fig. 4b).

The relative expression levels of ARG1 and NOS2 mRNAs in HLA-DR^-/low^ mononuclear cells from cancerous tissues were 1.79 times and 4.14 times higher, respectively, than those of paracancerous tissues (*p* < 0.01) (Fig. 4a); the relative expression levels of ARG1 and NOS2 protein were 2.2 times and 4.63 times higher, respectively, (*p* < 0.01) (n = 6) (Fig. 4c).

### **MDSCs from cancer patients suppressed both T cells proliferation and IFNγ production**

**Figure 5:**
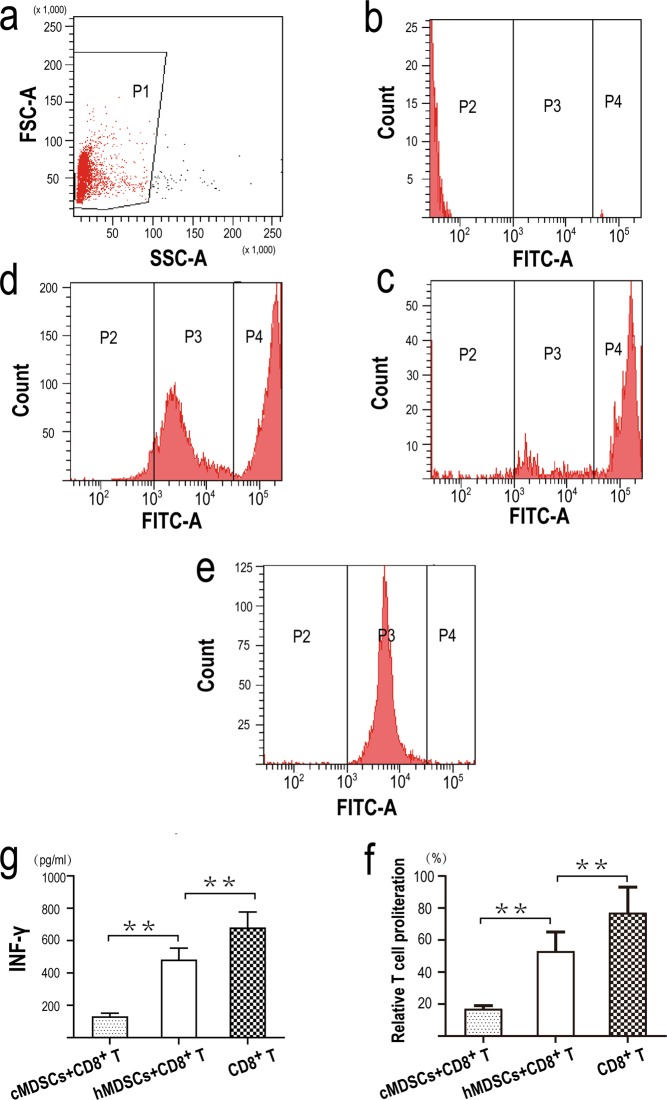
Functional analysis of MDSCs. **(a)** Cells were first gated based on FSC and SSC (population1, P1), subsequently, cells were gated based on the fluorescence intensity (P2, P3, P4) . **(b)** Flow cytometry chart of CD8^+^T cells-non labeled with CFSE. **(c)** Flow cytometry chart of CD8^+^T cell proliferation after 24 hour co-cultured with HLA-DR^-^CD33^+^MDSCs from cancer patients. **(d)** Flow cytometry chart of CD8^+^T cell proliferation after 24 hour co-cultured with HLA-DR^-^CD33^+^MDSCs from healthy donors. **(e)** Flow cytometry chart of CD8^+^T cell proliferation after 24 hour cultured alone. **(f, g)** HLA-DR^-^CD33^+^MDSCs from cancer patients inhibited both T cell proliferation**(f)** and IFNγ production(**g)**. For both graphs, Values were mean ± SEM (n = 6), **p < 0.01. *Abbreviations:cMDSCs, HLA-DR*^*-*^*CD33*^+^*MDSCs from cancer patients; hMDSCs, HLA-DR*^*-*^*CD33*^+^*MDSCs from healthy donors*.

HLA-DR^-^CD33^+^MDSCs from cancer patients or from healthy donors were co-cultured with CD8^+^T cells-labeled with CFSE respectively, and CD8^+^T cells were cultured alone. After 24 hour, CD8^+^T cell proliferation was analyzed on a CantoII flow cytometer(BD Biosciences). As shown in Fig. 5, compared to MDSCs from healthy donors, MDSCs from cancer patients suppressed both T cell proliferation and IFNγ production (p < 0.01) (Fig. 5f,g). At the same time, compared to a positive T cell proliferation control, MDSCs from healthy donors also suppressed both T cell proliferation and IFNγ production (p < 0.01) (Fig. 5f,g).

## Discussion

In 2000, a group reported the presence of CD11b^+^/Gr-1^+^myeloid progenitor cells in mice; These cells had the ability to inhibit CD8^+^T cells^28^. Subsequent studies have also found that in the peripheral blood of tumor patients, there is a type of immature myeloid cell that is devoid of lymphoid and mature markers, but with the ability to inhibit CD8^+^T cell response^29,30^. These cells were identified as immature myeloid cells^31,32^. As research in this field has progressed, some scholars have proposed that it would be more accurate to name such cells, myeloid-derived suppressor cells^33,34^.

MDSCs are a group of heterogeneous cells derived from IMCs. Under normal conditions, IMCs can be differentiated into the precursor of dendritic cells, macrophages and/or granulocytes. However, in the presence of tumors, inflammation, trauma and other conditions, the mature differentiation of IMCs is blocked and they transform into MDSCs^1^.

In mice, MDSCs can co-express myeloid differentiation antigen Gr1 and CD11b. According to the differences in expression levels of LY6G and LY6C (two different epitopes of Gr1), MDSCs can be divided into two subgroups: granulocytic MDSCs with a CDllb^+^LY6G^+^LY6C^low^ phenotype and monocytoid MDSCs with a CDllb^+^LY6G-LY6C^high^ phenotype^35,36^. In terms of human MDSCs, different authors have described different phenotypes, for example, HLA-DR^-^CD33^+^MDSCs^7,17,18,37^, Lin^-^HLA-DR^-^CD33^+^MDSCs^23–25,38^, Lin^-^HLA-DR^-^CD33^+^CD11b^+^CD15^+^MDSCs^38^, HLA-DR-CD33^+^CD11b^+^MDSCs^39,40^, HLA-DR^-^CD33^+^ CD11b^+^CD14^-^CD15^-^MDSCs^41,42^, Lin^-^HLA-DR^-^CD33^+^CD11b^+^MDSCs^43–45^, CD33^+^CD11b^+^ MDSC^46^, CD11b^+^CD14^-^CD15^+^/CD66b^+^MDSC^47^, CD14^-^CD15^+^MDSC^48^, HLA-DR^-/low^ CD14^+^MDSC^19–21,49–52^, HLA-DR^-/low^CD33^+^CD11b^+^CD14^+^MDSC^53–55^, HLA-DR^-/low^ CD14^+^CD15^-^^56^ and HLA-DR^-/low^ CD11b^+^CD14^+^^57^.

Some scholars believe that the MDSC subgroups can co-express the myeloid markers CD11b and CD33, but cannot express markers of mature myeloid cells, such as CD40, CD80, CD83 and HLA-DR. Instead, other researchers have proposed that HLA-DR^-/low^CD33^+^MDSCs can also be further sub-divided into monocytic MDSCs (HLA-DR^-/low^CD33^+^CD14^+^) and granulocytic MDSCs (HLA-DR^-/low^CD33^+^CD15^+^)^2,3^. Other researchers have classified human MDSCs as Lin^−^(CD3,CD14,CD16,CD19)HLA-DR^−^CD33^+^MDSCs, CD11b^+^CD14^−^CD33^+^ polymorphonuclear MDSCs and reported that PMN-MDSCs can also express CD15 and/or CD66b and that M-MDSCs are HLA-DR^−/low^CD14^+^M-MDSCs^13^. Another report proposed the naming and characterization standards of MDSCs as follows: CD11b^+^CD14^-^CD15^+^/CD11b^+^CD14^-^ CD66b^+^cells should be defined as PMN-MDSCs, while M-MDSCs are HLA-DR^-/low^ CD11b^+^CD14^+^CD15^-^M-MDSCs. Lin^-^(including CD3, CD14, CD15, CD19, CD56) HLA-DR^-^CD33^+^cells should be referred to as ‘early-stage MDSCs^15^.

MDSCs are derived through the action of a variety of cytokines secreted by various tumor and/or host cells, and as a result, the phenotype of MDSCs differs in different tumors. As yet, there are few *in vivo* studies of MDSCs in GC patients. Moreover, research has yet to elucidate the phenotypic characteristics of MDSCs under GC conditions, as well as the distribution and aggregation characteristics of MDSCs in patients, along with the activation of their immune suppression function. Furthermore, the relationship between the immune-avoidance of GC cells and MDSCs cannot be explained.

In related studies on gastric cancer, granulocytic MDSCs with CD15, CD66 and CD33 markers. and monocytic MDSCs with CD14 markers, have been found to increase significantly in the peripheral blood of patients. In other studies, Lin(CD3/CD19/CD56)^-^HLA-DR^-^CD33^+^MDSCs,

CD11b^+^CD15^+^CD14^-^MDSCs, CD15^+^FSC^hi^SSC^hi^MDSCs and CD14^+^HLA-DR^-^MDSCs were significantly increased in the PBMCs of GC patients^27,58^.

According to most literature, HLA-DR is not expressed by MDSCs, although the common myeloid markers, CD11b and CD33, are expressed. When we analyzed peripheral blood myeloid cells, we first selected HLA-DR/CD11b/CD33 for the immunophenotyping of peripheral blood myeloid cells. We also selected the granulocyte surface marker CD15, and the mononuclear surface marker CD14, for further immunophenotyping.

We divided the circulating myeloid cells into four phenotypes: HLA-DR^-^CD33^+^, HLA-DR^-^CD11b^+^, HLA-DR^-^CD15^+^ and HLA-DR^-^CD14^+^. First, we observed changes in the numbers of the four cell subtypes in peripheral blood under gastric cancer conditions. Compared with healthy volunteers, all four subtypes were significantly increased; the number of cells in each of the four subtypes was similar and did not differ significantly.

Further analysis of the expression level of ARG1 in different subtypes showed that the subtypes with high expression of ARG1 in the peripheral blood of healthy volunteers were mainly HLA-DR^-^ mononuclear cells, while the number of HLA-DR^-^ mononuclear cells with high expression levels of ARG1 in GC patients was significantly higher than that in healthy volunteers.

Our study suggested that although there was no difference in the number of the four subtypes, there were significant differences in the expression of ARG1. The expression frequency of ARG1 in HLA-DR^-^CD33^+^MDSCs was significantly higher than that in other MDSC subtypes, and the expression frequency of ARG1 in HLA-DR^-^CD14^+^MDSCs was also higher than that in HLA-DR^-^CD15^+^MDSCs and HLA-DR^-^CD11b^+^MDSCs, indicating that HLA-DR^-^CD33^+^MDSCs were different subtypes compared with the other three MDSC phenotypes. HLA-DR^-^CD14^+^MDSCs were not of the same type as HLA-DR^-^CD15^+^MDSCs and HLA-DR^-^CD11b^+^MDSCs, but there was no significant difference in the expression frequency of ARG1 between HLA-DR^-^CD15^+^MDSCs and HLA-DR^-^CD11b^+^MDSCs. Further immunophenotyping of HLA-DR^-^CD33^+^MDSCs was performed. We identified two main subgroups with high expression of ARG1 in the peripheral blood of healthy volunteers: HLA-DR^-^CD33^+^CD14^-^CD15^-^MDSCs and HLA-DR^-^CD33^+^CD14^-^CD15^+^MDSCs. While ARG1 was highly expressed only in the HLA-DR^-^CD33^+^CD14^-^CD15^-^MDSC subtype, accounting for more than 90%, the HLA-DR^-^CD33^+^CD14^-^CD15^+^subtype hardly expressed ARG1 in peripheral blood of GAC patients. These data gave us a new understanding of HLA-DR^-^CD33^+^MDSCs. The circulating HLA-DR^-^CD33^+^CD14^-^CD15^-^MDSCs under gastric cancer conditions are therefore the main force that plays an immunosuppressive role and such subtypes are early-stage MDSCs^7^.

It has been reported that the proportion of HLA-DR^-^CD14^+^MDSCs and HLA − DR^−^CD45^+^CD11b^+^CD14^+^MDSCs in GC tissues are significantly increased^20,59^. We therefore tried to find out whether other phenotypes of MDSCs exist in gastric cancer tissues. First, we analyzed the expression of ARG1 in HLA-DR^-^ mononuclear cells. Data indicated that the ARG1-expressing mononuclear cells are mainly the HLA-DR^-^ cells in GC tissue. Further immunophenotyping of HLA-DR^-^ mononuclear cells verified that the ARG1-expressing cells are derived mainly from HLA-DR^-^CD33^+^CD11b^-^, HLA-DR^-^CD33^+^CD11b^+^ and HLA-DR^-^CD33^-^CD11b^-^ subtype MDSCs, and that HLA-DR^-^CD33^-^CD11b^+^MDSCs barely express ARG1.

Further immunophenotyping of the first three MDSC subtypes verified that ARG1 was barely expressed in CD15^+^ granulocytic MDSCs, while ARG1 was highly expressed in over 35% of HLA-DR^-^CD33^-^CD11b^-^CD14^-^CD15^-^MDSCs. These subtype MDSCs lack a surface immunophenotype and have been tentatively named naïve MDSCs. Simultaneously, ARG1 was also highly expressed in more than 15% of HLA-DR^-^CD33^+^CD14^-^CD15^-^MDSCs, such subtypes are most likely early-stage MDSCs. Also, ARG1 was highly expressed in over 40% of HLA-DR^-^CD14^+^ monocytic MDSCs. On the whole, ARG1-expressing MDSCs were mainly naïve MDSCs, early-stage MDSCs and monocytic MDSCs in gastric cancer tissue. This study confirmed there were significant differences between circulating MDSCs and tumor-infiltrating MDSCs in terms of phenotypic characteristics and ARG1 expression under GC conditions.

Previous studies have shown that there is a large amount of ARG1 and NOS2 in MDSCs under tumor conditions and that these factors can inhibit the function of T cells, thereby creating an important mechanism for immune-avoidance^60,61^. We used RT-PCR and western blotting to determine the relative expression levels of ARG1 and NOS2 in HLA-DR^-^mononuclear cells in the peripheral blood and tumor tissues of GC patients. Data confirmed that there was a sufficiently high amount of ARG1 and NOS2 to cause T cell function inhibition in HLA-DR^-/low^ mononuclear cells under gastric cancer conditions.

Although our study highlights that phenotypic characteristics and the quantity of MDSCs, and the levels of ARG1, differ when compared between circulating and tumor-infiltrating MDSCs, our study was limited because there was not a large number of specimens and myeloid cell surface markers. In addition, this study did not focus on the specific mechanisms by which gastric cancer cells induce immunosuppression; these should be investigated in future research.

In Conclusion, our findings demonstrated for the first time that a large number of early-stage MDSCs can aggregate in the peripheral blood of GAC patients and express high levels of ARG1. We also verified that the specific subtypes of ARG1-expressing MDSCs in GAC tissue were mainly naïve MDSCs, early-stage MDSCs and monocytic MDSCs. There were significant differences in ARG1 expression and immunophenotype when comparing circulating MDSCs and tumor-infiltrating MDSCs. Therefore, we speculate that MDSCs aggregate in the peripheral blood and tumor tissues of GC patients and that this may represent an important mechanism by which gastric cancer cells induce immunosuppression. It is possible that this mechanism could explain the manner in which gastric cancer cells avoid the immune system.

## Supplementary information

Supplementary Dataset.

## Data Availability

All datasets on which the conclusions of the paper depend are available to readers.

## References

[1] Gabrilovich DI, Nagaraj S (2009). Myeloid-derived-suppressor cells as regulators of the immune system. Nat Rev Immunol..

[2] Talmadge JE, Gabrilovich DI (2013). History of myeloid-derived suppressor cells. Nat Rev Cancer..

[3] Khaled YS, Ammori BJ, Elkord E (2013). Myeloid-derived suppressor cells in cancer: recent progress and prospects. Immunol Cell Biol..

[4] Calcinotto A1, Spataro C1, Zagato E1, **et al**. IL-23 secreted by myeloid cells drives castration-resistant prostate cancer. Nature. **559**(7714):363–369(2018). 10.1038/s41586-018-0266-0 PMC6461206 29950727

[5] Pawelec G, Verschoor CP, Ostrand-Rosenberg S (2019). Myeloid-Derived Suppressor Cells: Not Only in Tumor Immunity. Front Immunol..

[6] Baert T (2019). Myeloid Derived Suppressor Cells: Key Drivers of Immunosuppression in Ovarian Cancer. Front Immunol..

[7] Greten TF, Manns MP, Korangy F (2011). Myeloid derived suppressor cells in human diseases. Int Immunopharmacol..

[8] Lu X (2017). Effective combinatorial immunotherapy for castration-resistant prostate cancer. Nature..

[9] Consonni FM (2019). Myeloid-Derived Suppressor Cells: Ductile Targets in Disease. Front Immunol..

[10] Yang L (2004). Expansion of myeloid immune suppressor Gr^+^CD11b^+^cells in tumor-bearing host directly promotes tumor angiogenesis. Cancer Cell..

[11] Liu C (2007). Expansion of spleen myeloid suppressor cells represses NK cell cytotoxicity in tumor-bearing host. Blood..

[12] Damuzzo V (2015). Complexity and challenges in defining myeloid-derived suppressor cells. Cytometry B Clin Cytom..

[13] Gabrilovich DI, Ostrand-Rosenberg S, Bronte V (2012). Coordinated regulation of myeloid cells by tumours. Nat Rev Immunol..

[14] Veglia F (2019). Fatty acid transport protein 2 reprograms neutrophils in cancer. Nature..

[15] Bronte V (2016). Recommendations for myeloid-derived suppressor cell nomenclature and characterization standards. Nat Commun..

[16] Uhel F (2019). Early-stage myeloid derived suppressor cell count: basophil exclusion matters. J Allergy Clin Immunol..

[17] Verschoor CP (2013). Blood CD33+HLA-DR-myeloid-derived suppressor cells are increased with age and a history of cancer. J Leukoc Biol..

[18] Lechner MG (2011). Functional characterization of human Cd33+ and Cd11b+myeloid-derived suppressor cell subsets induced from peripheral blood mononuclear cells co-cultured with a diverse set of human tumor cell lines. J Transl Med..

[19] Filipazzi P (2007). Identification of a new subset of myeloid suppressor cells in peripheral blood of melanoma patientswith modulation by a granulocyte-macrophage colony-stimulation factor-based antitumor vaccine. J Clin Oncol..

[20] Tian T (2015). Increased circulating CD14+HLA-DR-/low myeloid-derived suppressor cells are associated with poor prognosis in patients with small-cell lung cancer. Cancer Biomark..

[21] Li G (2015). Vasoactive intestinal peptide induces CD14^+^HLA-DR^-/low^ Myeloid-derived suppressor cells in gastric cancer. Mol Med Rep..

[22] Mengos AE, Gastineau DA, Gustafson MP (2019). The CD14+HLA-DR^lo/neg^ Monocyte: An Immunosuppressive Phenotype That Restrains Responses to Cancer Immunotherapy. Front Immunol..

[23] Javeed N (2016). Immunosuppressive CD14 + HLA-DR^lo/neg^ monocytes are elevated in pancreatic cancer and “primed” by tumor-derived exosomes. Oncoimmunology..

[24] Shen P (2014). Increased circulating Lin^-/low^ CD33+ HLA-DR- myeloid-derived suppressor cells in hepatocellular carcinoma patients. Hepatol Res..

[25] Toor SM (2017). Myeloid cells in circulation and tumor microenvironment of breast cancer patients. Cancer Immunol Immunother..

[26] Kusmartsev S (2008). Reversal of myeloid cell mediated immunosuppression in patients with metastatic renal cell carcinoma. Clin Cancer Res..

[27] Duffy A (2013). Comparative analysis of monocytic and granulocytic myeloid-derived suppressor cell subsets in patients with gastrointestinal malignancies. Cancer Immunol Immunother..

[28] Wang L (2013). Increased myeloid-derived suppressor cells in gastric cancer correlate with cancer stage and plasma S100A8/A9 proinflammatory proteins. J Immunol. 15.

[29] Bronte V (2000). Identification of a CD11b( + )/Gr-1(+)/CD31( + ) myeloid progenitor capable of activating or suppressing CD8( + ) T cells. Blood..

[30] Bronte V (2001). Tumor-induced immune dysfunctions caused by myeloid suppressor cells. J Immunother..

[31] Almand B (2001). Increased production of immature myeloid cells in cancer patients: A mechanism of immunosuppression in cancer. J Immunol..

[32] Kusmartsev S, Gabrilovich DI (2002). Immature myeloid cells and cancer associated immune suppression. Cancer Immunol Immunother..

[33] Mirza N (2006). All-trans-retinoic acid improves differentiation of myeloid cells and immune response in cancer patients. Cancer Res..

[34] Gabrilovich DI (2007). The terminology issue for myeloid-derived suppressor cells. Cancer Res..

[35] Krystal G (2007). The terminology issue for myeloid-derived suppressor cells. Cancer Res..

[36] Youn JI (2008). Subsets of myeloid-derived suppressor cells in tumor-bearing mice. J.Immunol..

[37] Peranzoni E (2010). Myeloid-derived suppressor cell heterogeneity and subset definition. Curr Opin Immunol..

[38] Bartmann C (2016). CD33(+)/HLA-DR(neg) and CD33( + ) /HLA-DR( + /-) Cells: Rare populations in the human decidua with characteristics of MDSC. Am J Reprod Immunol..

[39] Khaled YS, Ammori BJ, Elkord E (2014). Increased levels of granulocytic myeloid-derived suppressor cells in peripheral blood and tumour tissue of pancreatic cancer patients. J Immunol Res..

[40] Sade-Feldman M (2016). Clinical Significance of Circulating CD33 + CD11b + HLA-DR-Myeloid Cells in Patients with Stage IV Melanoma Treated with Ipilimumab. Clin. Cancer Res..

[41] Zhang H (2015). Myeloid-derived suppressor cells inhibit T cell proliferation in human extranodal NK/T cell ymphoma: a novel prognostic indicator. Cancer Immunol Immunother..

[42] Toor SM (2016). Increased levels of circulating and tumor-infiltrating granulocytic myeloid cells in colorectal cancer patients. Front Immunol..

[43] Raychaudhuri B (2015). Myeloid derived suppressor cell infiltration of murine and human gliomas is associated with reduction of tumor infiltrating lymphocytes. J Neurooncol..

[44] Diaz-Montero CM (2009). Increased circulating myeloid-derived suppressor cells correlate with clinical cancer stage, metastatic tumor burden, and doxorubicin –cyclophosphamide chemotherapy. Cancer Immunol Immunother..

[45] Scrimini S (2015). Expansion of myeloid-derived suppressor cells in chronic obstructive pulmonary disease and lung cancer: potential link between inflammation and cancer. Cancer Immunol Immunother..

[46] Salem ML (2017). IFN-α-based treatment of patients with chronic HCV show increased levels of cells with myeloid-derived suppressor cell phenotype and of IDO and NOS. Immunopharmacol Immunotoxicol..

[47] Fernandez IE (2016). Peripheral blood myeloid-derived suppressor cells reflect disease status in idiopathic pulmonary fibrosis. Eur Respir J..

[48] Dumitru CA (2012). Neutrophils and granulocytic myeloid-derived suppressor cells: immunophenotyping, cell biology and clinical relevance in human oncology. Cancer Immunol Immunother..

[49] Uhel F (2017). Early expansion of circulating granulocytic myeloid-derived suppressor cells predicts development of nosocomial infections in patients with sepsis. Am J Respir Crit Care Med..

[50] Poschke I (2010). Immature Immunosuppressive CD14 + HLA-DR-/low Cells in Melanoma Patients Are Stat3hi and Overexpress CD80,CD83,and DC-Sign. Cancer Res..

[51] Zhang ZJ (2015). Immune independent crosstalk between lymphoma and myeloid suppressor CD14+HLA-DR^low/neg^ monocytes mediates chemotherapy resistance. Oncoimmunol..

[52] Du J, Sun X, Song Y (2017). The study of CD14 + HLA-DR-/low myeloid-drived suppressor cell (MDSC) in peripheral blood of peripheral T-cell lymphoma patients and its biological function. Cell Mol Biol (Noisy-le-grand)..

[53] Rudolph BM (2014). Increased frequencies of CD11b( + ) CD33( + ) CD14( + ) HLA-DR(low)myeloid-derived suppressor cells are an early event in melanoma patients. Exp Dermatol..

[54] Bao Y, Mo J, Ruan L, Li G (2015). Increased monocytic CD14^+^HLADRlow/-myeloid-derived suppressor cells in obesity. Mol Med Rep..

[55] Ren JP (2016). Hepatitis C virus-induced myeloid-derived suppressor cells regulate T-cell differentiation and function via the signal transducer and activator of transcription 3 pathway. Immunol..

[56] Bernsmeier C (2018). CD14 + CD15-HLA-DR-myeloid-derived suppressor cells impair antimicrobial responses in patients with acute-on-chronic liver failure. Gut..

[57] Chen MF (2014). IL-6-stimulated CD11b + CD14 + HLA-DR-myeloid-derived suppressor cells, are associated with progression and poor prognosis in squamous cell carcinoma of the esophagus. Oncotarget..

[58] Mesali H (2016). Regulatory T Cells and Myeloid-Derived Suppressor Cells in Patients with Peptic Ulcer and Gastric Cancer. Iran J Immunol..

[59] Choi HS (2016). The prognostic effects of tumor infiltrating regulatory T cells and myeloid derived suppressor cells assessed by multicolor flow cytometry in gastric cancer patients. Oncotarget..

[60] Zea AH (2005). Arginase-producing myeloid suppressor cells in renal cell carcinoma patients: a mechanism of tumor evasion. Cancer Res..

[61] Paulo C (2009). Arginase I–Producing Myeloid-Derived Suppressor Cells in Renal Cell Carcinoma Are a Subpopulation of Activated Granulocytes. Cancer Res..

