# Supplementary material for: Circulating and tumor-infiltrating arginase 1-expressing cells in gastric adenocarcinoma patients were mainly immature and monocytic Myeloid-derived suppressor cells
Source: Sci Rep. 2020 May 15;10:8056. doi: 10.1038/s41598-020-64841-4 (PMC7229115; doi:10.1038/s41598-020-64841-4)
Supplement: Supplementary file 1 — Supplementary Dataset. [file 41598_2020_64841_MOESM1_ESM.docx]

### Circulating and tumor-infiltrating arginase1-expressing cells in gastric adenocarcinoma patients were mainly immature and monocytic Myeloid-derived suppressor cells

WeiHong Ren^1^*, XuRan Zhang^1^, WenBo Li^1^, Qian Feng^1^, HuiJie Feng^1^, Yan Tong^1^, Hao Rong^1^, Wei Wang^1^, Dai Zhang^1^, ZhenQiang Zhang^2^ and ShiChun Tu^3^

**Supplementary Figure 1. Isotype control chart for figure 2**

Immune cells were labeled with isotype fluorescent antibodies (mouse IgG1-FITC/mouse IgG2b-PE/mouse IgG1-APC/rat IgG2b APC-CY7/mouse IgG2a-PerCP-Cy5.5/mouse IgM-PE-CY7) (BD Biosciences) respectively. The cells were gated based on isotype control, the count of positive cells < 0.2% by flow cytometry. As shown in supplementary figure 1, the panel of SSC-HLA-DR-isotype**(a)**, HLA-DR-Arg1-isotype**(b)**, CD11b-Arg1-isotype**(c)**, CD14-Arg1-isotype**(d)**, CD33-Arg1-isotype**(e)** and CD15-Arg1-isotype**(f)**.

**Supplementary Figure 2. Full length blots for figure 4**

**(a)** The relative expression levels of ARG1 and NOS2 in the circulating HLA-DR^-/low^ mononuclear cells (n=20). **(b)** The relative expression levels of ARG1 and NOS2 in the tumor-infiltrating HLA-DR^-/low^ mononuclear cells (n=6). *Abbreviations: GC-PB, gastric cancer-periferal blood; HD-PB, healthy donor-periferal blood. GC-T, gastric cancer-tissue; PC-T, para-carcinoma-tissues. Data in A and B are the representative of three independent experiments.*

**Supplementary Table 1. Backgrounds of gastric cancer patients and volunters**

20 peripheral blood samples was collected from gastric adenocarcinoma patients prior to surgery, Simultaneously, 20 volunter’s samples was collected. backgrounds of Patients and volunteers were described in Supplemental Table1.

**Supplementary Fig 1**

**
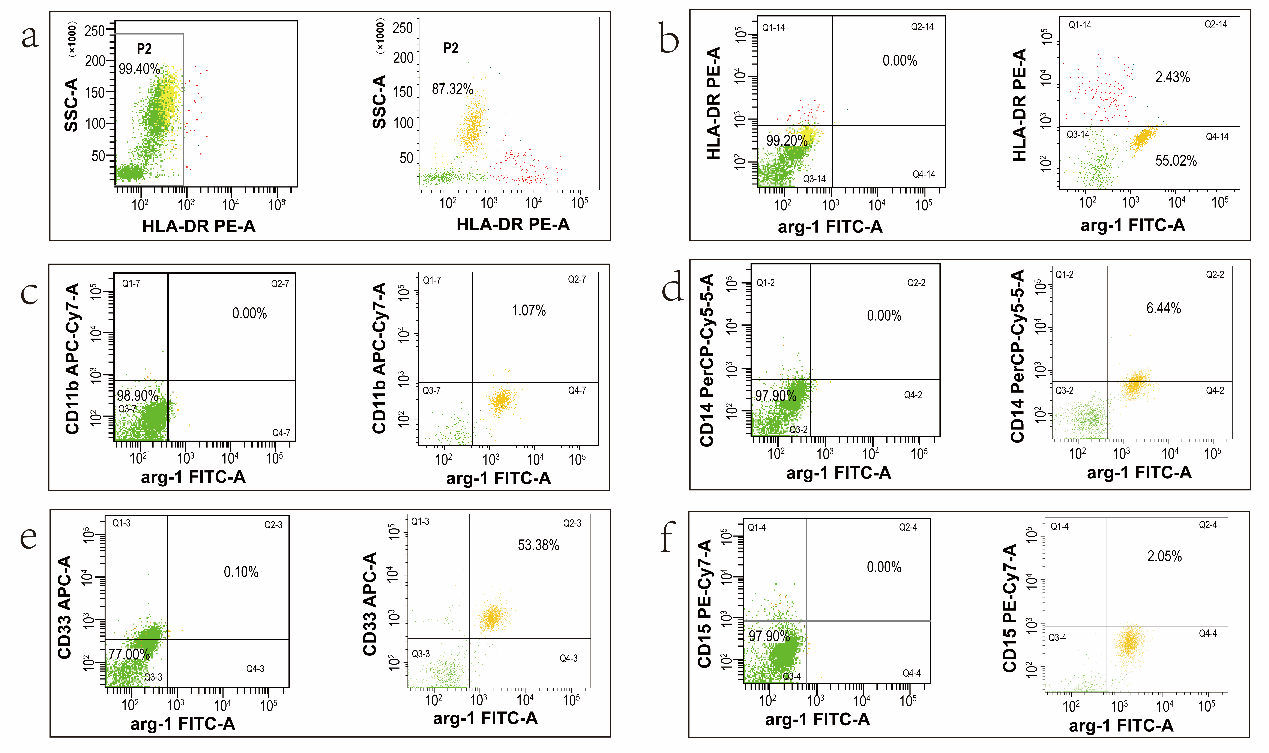
**


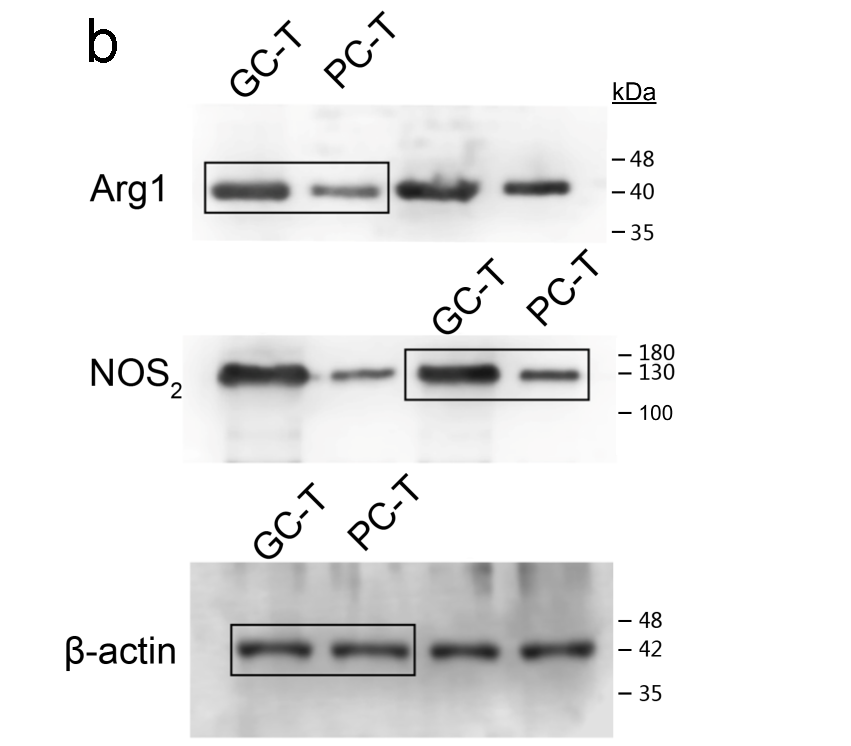

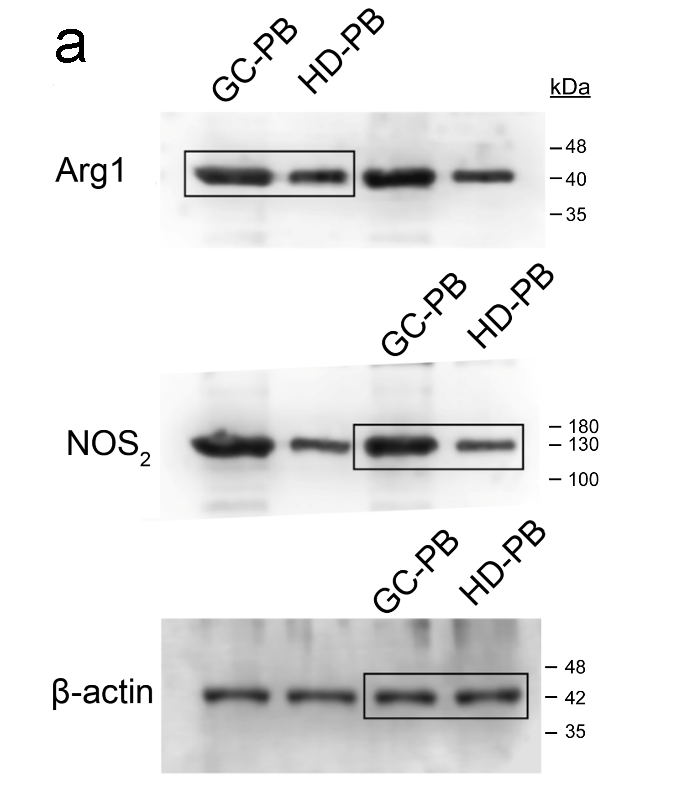
 **Supplementary Fig 2**

**Supplementary Table1**

Backgrounds of gastric cancer patients and volunters

| Clinical parameters patients set (n = 20) volunters set (n = 20) |
| --- |
| Age (years)  <30 1 1  >30 and <60 8 8  >60 11 11  Male 14 14  Female 6 6 |
